# An Image Registration Method Based on Correlation Matching of Dominant Scatters for Distributed Array ISAR

**DOI:** 10.3390/s22041681

**Published:** 2022-02-21

**Authors:** Liqi Zhang, Yanlei Li

**Affiliations:** 1 University of Chinese Academy of Sciences, Beijing 100049, China; zhangliqi19@mails.ucas.ac.cn; 2 National Key Laboratory of Microwave Imaging Technology, Aerospace Information Research Institute, Chinese Academy of Sciences, Beijing 100190, China

**Keywords:** distributed array ISAR, image registration, SIFT, correlation matching, three-dimensional imaging

## Abstract

Distributed array radar provides new prospects for three-dimensional (3D) inverse synthetic aperture radar (ISAR) imaging. The accuracy of image registration, as an essential part of 3D ISAR imaging, affects the performance of 3D reconstruction. In this paper, the imaging process of distributed array ISAR is proposed according to the imaging model. The ISAR images of distributed array radar at different APCs have different distribution of scatters. When the local distribution of scatters for the same target are quite different, the performance of the existing ISAR image registration methods may not be optimal. Therefore, an image registration method is proposed by integrating the feature-based method and the area-based method. The proposed method consists of two stages: coarse registration and fine registration. In the first stage, a dominant scatters model is established based on scale-invariant feature transform (SIFT). In the second stage, sub-pixel precision registration is achieved using the local correlation matching method. The effectiveness of the proposed method is verified by comparison with other image registration methods. The 3D reconstruction of the registered experimental data is carried out to assess the practicability of the proposed method.

## 1. Introduction

Inverse synthetic aperture radar (ISAR) is a type of radar used for the imaging of noncooperative moving targets. ISAR transmits wideband signals to obtain high-range resolution and achieves high azimuth resolution via synthetic aperture caused by relative motions [1]. ISAR technology enables radar systems to develop from target detection and ranging [2,3,4] to acquiring detailed features of the target [5,6,7]. Traditional ISAR imaging is the two-dimensional (2D) projection of the target onto the imaging plane, which only reflects the shape information of the target. In addition, 2D ISAR imaging suffers from problems such as information loss and image feature instability. A high-resolution three-dimensional (3D) image can reflect the 3D geometry of the target and provide more robust features.

Earlier researchers mainly used a single-station wideband radar to implement 3D ISAR imaging. The main methods include 3D imaging based on sum-diff beams [8,9] and 3D reconstruction based on sequence ISAR images [10,11]. The sequential ISAR imaging method is highly sensitive to the target’s motion posture, causing difficulties in ensuring the accuracy of the 3D reconstruction result, and thus limited practical value. Therefore, researchers have focused on multiview 3D ISAR imaging technology; multi-station radars are used to observe the target at the same time to obtain 3D images. In recent years, many 3D ISAR imaging methods, such as interferometric ISAR imaging [12,13,14], array ISAR [15,16] and MIMO ISAR [17], have been studied by scholars. Among these, distributed array radar combining MIMO radar real-aperture imaging and ISAR synthetic-aperture imaging has become a research hotspot, as it can shorten the imaging time and obtain a higher elevation resolution. In particular, distributed array ISAR imaging requires high-quality 2D ISAR images from each antenna. After image registration, a spectral analysis is conducted on the 2D ISAR images with different antenna phase centers (APCs) to obtain the 3D reconstruction results.

One of the keys to 3D ISAR imaging technology is the multiview 2D ISAR image registration, which is primarily achieved by area-based, feature-based or hybrid methods. Area-based methods focus on image intensity, including the correlation matching method [18] and the max-spectrum method [19]. In the case of a short-length baseline, the correlation matching method and the max-spectrum method are commonly used for ISAR image registration. Feature-based methods focus on feature extraction. These methods include the Harris corner detector method [20], the scale-invariant feature transform (SIFT) method and its improved versions [21,22,23,24]. A SIFT-like algorithm was first proposed in [25] for multiview SAR image registration. An automatic and fast image registration method was presented in [26] for GF-3 SAR images, which combined an adaptive sampling method with the SAR-SIFT algorithm. In [27], an improved SIFT method was proposed for single-station sequential ISAR image registration, and 3D reconstruction of the simulated aircraft data was carried out. However, distributions of scatters of the same target on different APCs are different due to the long baseline length of the distributed array antenna. Overall, area-based methods are subject to image mismatch, while feature-based methods suffer from insufficient registration accuracy [28].

Thus far, there is in general very little research that has been conducted on distributed array ISAR image registration. Inspired by the application of the SAR-SIFT algorithm in multiview SAR image registration, this paper proposes an image registration method that combines the advantages of feature-based and area-based methods to address the above problems. Our main contributions include:

1. Based on the SAR-SIFT algorithm, a dominant scatters model is proposed for multi-view ISAR image registration;
2. Compared with existing ISAR image registration methods, the superiority of the proposed method is verified;
3. Subpixel registration and 3D reconstruction are carried out on different experimental data to verify the effectiveness and practicability of the proposed method.

The rest of this paper is organized as follows. Section 2 outlines the 3D imaging model of the distributed array ISAR system. Our proposed image registration algorithm based on dominant scatters is described in Section 3. In Section 4, the registration analysis of different registration methods on the experimental data is presented, and the 3D reconstruction is carried out. Finally, the conclusions are drawn in Section 5.

## 2. Imaging Model of the Distributed Array ISAR System

**Figure 1:**
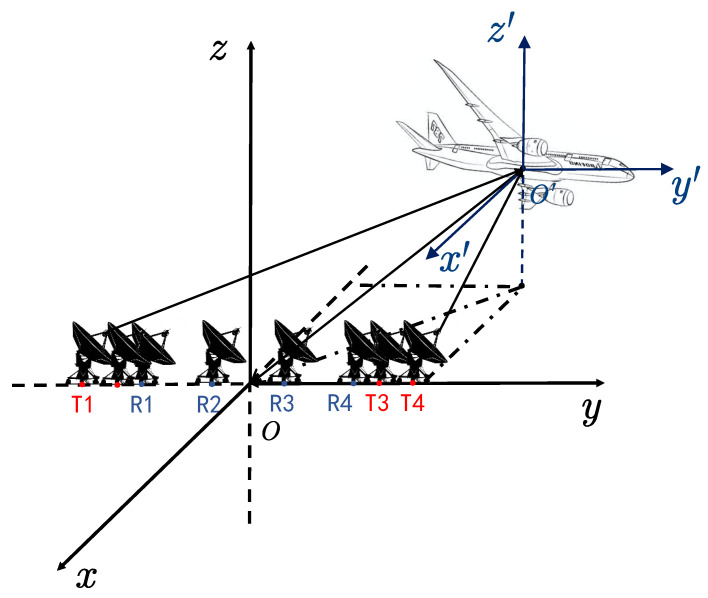
A geometric overview of the distributed array ISAR imaging.

Figure 1 shows the geometry of the distributed array ISAR for 3D ISAR imaging. The radar system consists of a central station (CO), N transmitting stations with the same structure (Tx) and N receiving stations with the same structure (Rx), which means that there are Np (=N×N) APCs. Here, we assume that the target bears a constant velocity in the direction of ***v***.

The echo signal is denoted by deskewing:

(1) $$\begin{array}{l} S_{Rm}(t)=V_{Rm}\times \mathrm{rect}\left[\frac{t-\tau_{m}+\Delta \tau_{m}/2}{T_{p}-\Delta \tau_{m}}\right]\times \\ \cos[2\pi k_{RF}\Delta \tau_{m}(t-\tau )+2\pi f_{RF}\Delta \tau_{m}(t-\tau )+\pi k_{RF}\Delta \tau_{m}^{2}] \end{array}$$

where $\Delta \tau_{m}$ is the delay experienced by the radar signal from the transmitting antenna to the receiving antenna through the target and $V_{Rm}$, $k_{RF}$ and $T_{p}$ denote the amplitude, chirp rate and period of the echo signal, respectively.

A Fourier transform is applied after sampling the output deskew signal. This gives rise to the result of the range dimension pulse compression, expressed as follows:

(2) $$\begin{array}{l} I_{Rm}(f)=A_{Rm}\sin c[(T_{p}-\Delta \tau m)(f-k_{RF}\Delta \tau_{m})]\times \\ \exp[j(2\pi f_{RF}\Delta \tau_{m}+\Delta \varphi_{m})] \end{array}$$

where $\Delta \varphi_{m}$ is the additional phase introduced into the signal transmission.

With the N×N APCs each being an independent channel, Np-channel 2D ISAR images are obtained by motion compensation after applying $S_{c}(f)=\exp(-j\Delta \varphi )$ for the additional phase as follows:

(3) $$\begin{array}{l} I_{np}(f,f_{m})=A_{i}\sin c\left\{T_{p}{}^{\prime }[f+\frac{k_{RF}}{c}(R_{Tn}+R_{Rm}-2R_{ref})]\right\}\times \\ \sin c\left\{T_{A}[f_{m}+\frac{\left(V_{Tn}+V_{Rm}\right)}{\lambda }]\right\}\times \\ \exp\left\{-j\frac{2\pi f_{RF}}{c}(R_{Tn}+R_{Rm}-2R_{ref})\right\} \end{array}$$

where n is the index of the transmitting antenna, m is the index of the receiving antenna, $R_{Tn}$ and $R_{Rm}$ are the distance from the target to the n-th transmitting antenna and m-th receiving antenna, respectively, $V_{Tn}$ and $V_{Rm}$ are the target’s velocity relative to the n-th transmitting antenna and the m-th receiving antenna, respectively, $f_{m}$ is the Doppler frequency and λ is the wavelength corresponding to the center frequency of the radar’s transmitted signal.

**Figure 2:**
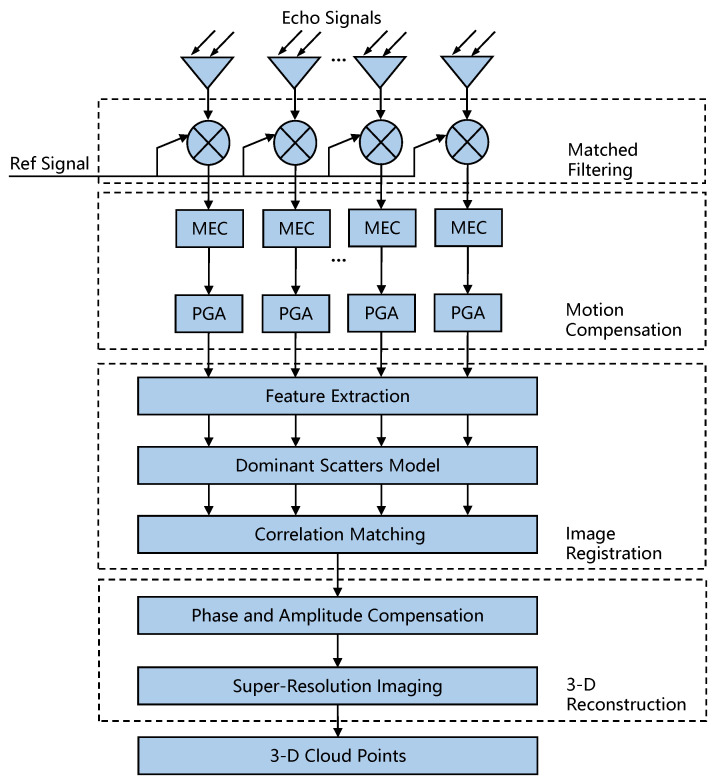
Flowchart of the imaging process of the distributed array ISAR.

Figure 2 presents a flowchart of the imaging process of the distributed array ISAR. The 16 echo signals of different APCs are passed through matched filtering, motion compensation and other signal processing steps to obtain 16 ISAR images. Among them, motion compensation mainly solves the problem of envelope shift caused by translational motion and high-order phase error caused by rotational motion. Next, the proposed method is used to register multiview ISAR images, and the registration area in the master image is used as the reference calibration area to correct the amplitude and phase errors. Finally, through super-resolution imaging processing of the elevation dimension, 3D re-construction of the target is realized.

## 3. ISAR Image Registration Method Based on Correlation Matching of Dominant Scatters

**Figure 3:**
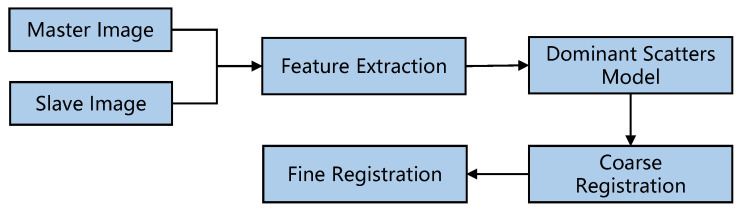
Flowchart of the proposed image registration method.

Following the imaging model introduced in Section 2, the proposed image registration method for a distributed array radar system is proposed in this section. Firstly, the SIFT algorithm is used to extract the features between the master image and the slave images, and the random sample consensus (RANSAC) algorithm is adopted to eliminate the mismatched relationship. Then, the registration control points are determined by the dominant scatters model. Finally, the relative offset is determined via correlation matching to complete image registration. The flowchart of the proposed method is shown in Figure 3.

### 3.1. Feature Extraction

SIFT is an algorithm for detecting and describing local features in images; it is widely adopted in the field of computer vision. First proposed by David Lowe in 1999 and subsequently supplemented and improved in 2004, SIFT adopts a Gaussian convolution kernel for scale transformation to obtain the corresponding scale space of the image, which can be calculated by the following expression [21]:

(4) L(x,y,σ)=G(x,y,σ)⊗I(x,y)

where σ is the scale space factor, (x,y) are the pixel coordinates of the image at the scale of σ and ⊗ denotes the convolution operation in the *x* and *y* directions. The Gaussian convolution kernel G(x,y,σ) is given by:

(5) $$G(x,y,\sigma )=\frac{1}{2\pi \sigma^{2}}e^{-\frac{x^{2}+y^{2}}{2\sigma^{2}}}$$

The difference-of-Gaussian scale space is computed by convolving the difference-of-Gaussian kernel of different scales with the image:

(6) $$\begin{array}{l} D(x,y,\sigma )=[G(x,y,\eta \sigma )-G(x,y,\sigma )]⊗I(x,y)\\ =L(x,y,\eta \sigma )-L(x,y,\sigma ) \end{array}$$

The image is sampled at different scales to improve the anti-noise performance of feature extraction, while achieving the invariant transformation of the image scales. The extrema of the scale space are acquired from the difference images of adjacent images in the same frequency order, and the exact feature points are obtained by 2D function fitting. Next, the gradient directions with high robustness are computed using the statistical properties of the image’s gradient direction histogram around the feature points. The calculated gradient directions are then used as the main direction of the feature points. The magnitude and direction of the corresponding gradient at (x,y) are given by:

(7) $$\begin{array}{l} m(x,y)=\sqrt{{\left(L(x+1,y)-L(x-1,y)\right)}^{2}+{\left(L(x,y+1)-L(x,y-1)\right)}^{2}}\\ \theta (x,y)=\arctan\left(\frac{L(x,y+1)-L(x,y-1)}{L(x+1,y)-L(x+1,y)}\right) \end{array}$$

Treating the image pixel as a unit, for the current feature point, the main direction of the feature point is obtained by using the statistical characteristics of the gradient histogram of the region around the extracted feature point. First, we rotate the axes to the orientation of the feature points to ensure rotation invariance. Next, we take a 16 × 16 window centered on the feature point. Then, eight gradient direction histograms are calculated on a 4 × 4 small block, and the accumulated value of each gradient direction is drawn to form a seed point. Each feature point is described by 4 × 4 = 16 seed points; each seed point has the information of eight direction vectors. Finally, a 16 × 8 = 128-dimensional SIFT feature vector is obtained.

**Figure 4:**
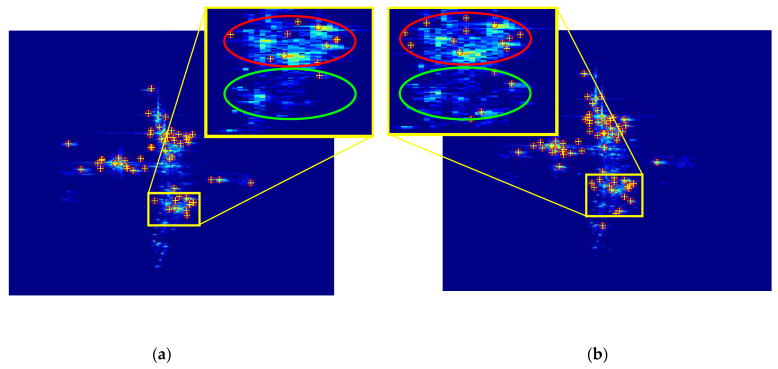
The experimental ISAR 2D images. (**a**) The master image with 68 feature points selected. (**b**) The slave image with 92 feature points selected.

The 2D ISAR image of the distributed array ISAR is mainly composed of dominant scatters. Figure 4 shows the distribution of scatters in the master image and the slave image in different viewpoints. The engine of the target is selected for zoom-in analysis. The dominant scatters in the red circle have relatively robust characteristics, while the same target has different scatter characteristics in different viewpoints in the green circle. It can be seen that the feature point correspondence between the master image and the slave image is unstable after feature extraction with SIFT.

The feature points include corner points, edge points, bright spots in dark areas and dark points in bright areas, all of which are not directly relevant to image registration. Generally speaking, dominant scatters are used as registration control points in ISAR image registration. After feature extraction, the RANSAC algorithm is leveraged to establish a dominant scatters model to determine registration control points through mapping relations.

### 3.2. Improved Correlation Matching Method Based on Dominant Scatters

#### 3.2.1. Dominant Scatters Model

For the master image and the slave images, SIFT obtains two groups of descriptor vectors. If the element of two descriptor vectors has a Euclidean distance greater than a certain threshold, the element is selected as a feature point. The Euclidean distance corresponding to the descriptor vectors of two groups of different feature points is computed as follows [29]:

(8) $$\begin{array}{l} Dis_{i,j}(\varepsilon )=\left\|X_{i,\varepsilon }^{1}-X_{j,\varepsilon }^{2}\right\|\\ =\sqrt{{\left(x_{i,\varepsilon }^{1}-x_{j,\varepsilon }^{2}\right)}^{2}+{\left(y_{i,\varepsilon }^{1}-y_{j,\varepsilon }^{2}\right)}^{2}} \end{array}$$

Based on the geometric similarity between feature points in the master image and the slave images, a dominant scatters model between different 2D images is established. RANSAC denotes the correct matching points as inner points and the incorrect matching points as outer points. The steps of the RANSAC algorithm are shown in Table 1. The parameters of the model are estimated iteratively from a group of observed data containing the incorrect matching points.

The Euclidean distance corresponding to two sets of descriptor vectors of different feature points is taken as the dataset, and the probability of the interior point in the whole dataset is assumed to be ϖ, given by:

(9) $$\varpi =\frac{n_{inliers}}{n_{inliers}+n_{outliers}}$$

Assuming that two points are required to determine the above model and K is the number of iterations, then the probability of obtaining the correct solution is as follows:

(10) $$P=1-{\left(1-\varpi^{n}\right)}^{K}$$

According to Equations (9) and (10), the number of iterations K is defined as:

(11) $$K=\frac{\log(1-P)}{\log(1-\varpi^{n})}$$

#### 3.2.2. Calculating the Relative Offset

The proposed registration method involves two steps, namely coarse registration and fine registration. Based on the coarse registration, the value of the selected control point is interpolated, and the correlation matching method is used to perform fine registration with the subpixel unit. Through these steps, the registration accuracy is expected to reach the subpixel level.

The first step is coarse registration of the gridded master image, and the extremum in the grid is taken as the alternative control point. SIFT extracts feature points for corner points. The position of the alternative control points in the slave image is determined by the dominant scatters model described in the above section, and the alternative control points with high correlation between images are selected as the control points to reach the pixel registration accuracy.

The second step is fine registration. A matching window around the control points is taken to perform 16-fold 2D linear interpolation to meet the subpixel registration accuracy. We set the two matching windows to be registered to be $A_{1}(i,j)$ and $A_{2}(i,j)$. Then, the normalized cross-correlation function of the two images can be obtained:

(12) $$\begin{array}{l} Xcorr=\frac{\sum_{(i,j)\in W}\left|\phi_{1}(i,j)\times \phi_{2}(i+r,j+c)\right|}{\sqrt{\sum_{(i,j)\in W}\phi_{1}{\left(i,j\right)}^{2}\times \sum_{(i,j)\in W}\phi_{2}{\left(i+r,j+c\right)}^{2}}}\\ \phi_{k}(i,j)=A_{k}(i,j)-E[A_{k}(i,j)] \end{array}$$

where r and c represent the offset of the row and column directions of the two images, respectively, W is the size of the image area and $\begin{array}{l} E[*] \end{array}$ is the mean value of the image.

When the master image and the slave image are accurately registered, the relative offset of the center position of the correlation coefficient window is effectively the accurate offset of registration. This method can also be implemented in the frequency domain by Fourier transform.

Once the exact offset of the corresponding point is obtained, the polynomial model is employed for accurate correction. The polynomial model establishes a set relation between radar image coordinates and the target’s physical coordinates. It considers the global deformation of a 2D ISAR image as the resultant effect of translation, scaling, rotation and other higher deformations. The binary quadratic polynomial is as follows:

(13) $$\begin{array}{l} {x^{\prime }}_{k}=a_{k0}+a_{k1}x+a_{k2}y+a_{k3}x^{2}+a_{k4}y^{2}+a_{k5}xy\\ {y^{\prime }}_{k}=b_{k0}+b_{k1}x+b_{k2}y+b_{k3}x^{2}+b_{k4}y^{2}+b_{k5}xy \end{array}$$

According to Equation (12), an offset is calculated in the master image $A_{1}(i,j)$. Its matching position is then found in the slave image $A_{2}(i,j)$. The value at this position is obtained by using bilinear interpolation to interpolate the complex number of pixels around the matching position in the slave image $A_{2}(i,j)$.

The bilinear interpolation is expressed as:

(14) $$\begin{array}{l} I(P)=\sum_{i=1}^{2}\sum_{j=1}^{2}I(i,j)\times W(i,j)\\ =\left[\begin{array}{cc} I_{11} & I_{12}\\ I_{21} & I_{22} \end{array}\right]\times \left[\begin{array}{cc} W_{11} & W_{12}\\ W_{21} & W_{22} \end{array}\right] \end{array}$$

where $W_{ij}=W(x_{i})W(y_{j})$.

(15) $$\begin{array}{l} W(x_{1})=1-\Delta x;W(x_{2})=\Delta x\\ W(y_{1})=1-\Delta y;W(y_{2})=\Delta y \end{array}$$

Substituting Equation (15) into Equation (14) yields:

(16) $$\begin{array}{l} I(P)=W_{11}I_{11}+W_{12}I_{12}+W_{21}I_{21}+W_{22}I_{22}\\ =(1-\Delta x)(1-\Delta y)I_{11}+(1-\Delta x)\Delta yI_{12}+\Delta x(1-\Delta y)I_{21}+\Delta x\Delta yI_{22} \end{array}$$

The slave image is registered with the master image after bilinear interpolation.

## 4. Experimental Results

In this section, the performance of the proposed method is analyzed by using experimental data collected from distributed array radars. In Section 4.1, an experiment is described in which we obtained multiview 2D ISAR images of different channels. The registration details of the proposed method are also presented. The analysis and comparison of different registration methods for 16-channel 2D ISAR images are given in Section 4.2. Section 4.3 presents the 3D ISAR imaging results for a variety of aircraft based on 16-channel 2D ISAR images after registration, verifying the practicality of the proposed method.

### 4.1. Distributed Array Radar System and Image Registration

**Figure 5:**
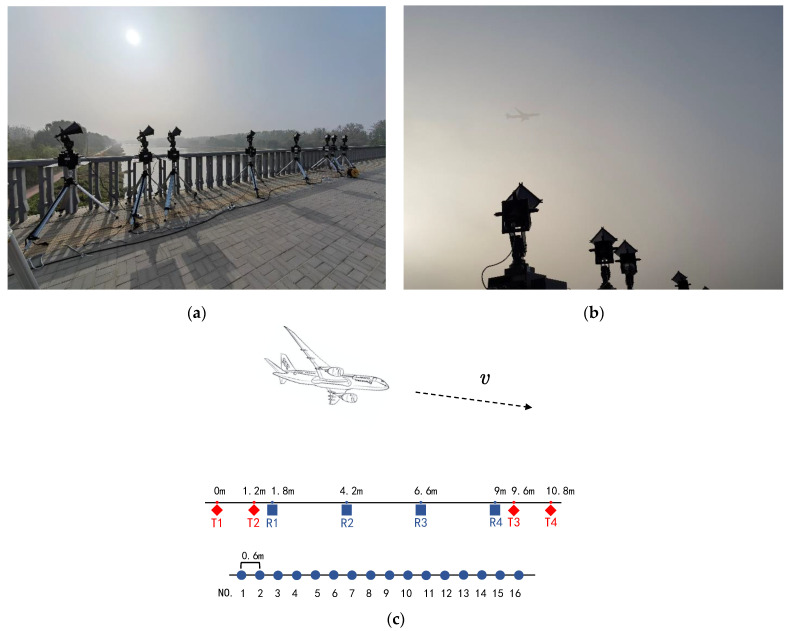
The radar system and our experiment setup. (**a**) The distributed array ISAR system. (**b**) The observed airplane. (**c**) Distribution of APCs. The distance between adjacent array elements is d. The maximal baseline length is D.

To assess the effectiveness of the proposed method, the ISAR imaging experiment of the distributed array radar was carried out near the Beijing Capital International Airport. Relevant system parameters are listed in Table 2. Figure 5 shows a distributed array radar system and the observation aircraft in foggy weather conditions. The system consists of four transmitters and four receivers.

**Figure 6:**
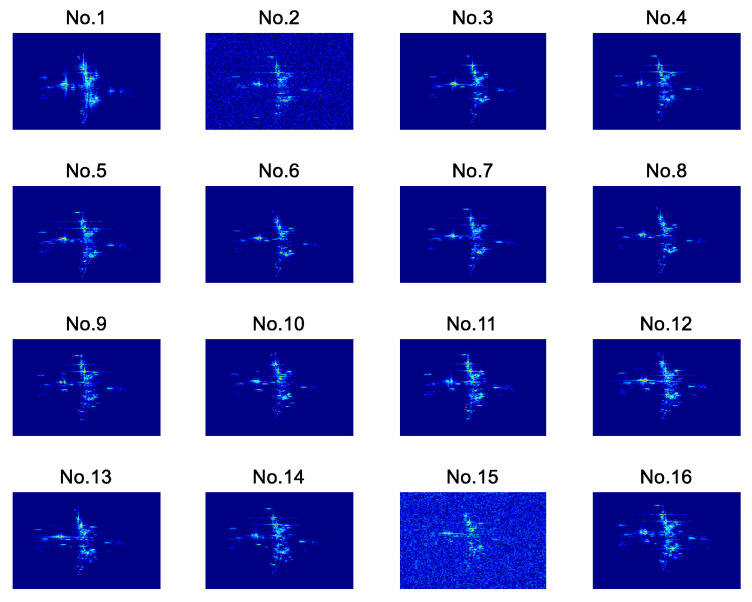
Examples of 16-channel 2D ISAR images. The channel-2 image and channel-15 image are with a lower SNR. The radar cross-section (RCS) characteristics of each channel 2D image are inconsistent in different viewpoints.

The 16-channel echo signals are obtained through a reasonable layout of the equipment. As shown in Figure 6, a total of 16 2D ISAR images are obtained after 2D ISAR imaging of the 16-channel echo signals. The difference in the location of the transceiver antennas leads to the imaging planes of the target not coinciding with each other. The 2D ISAR images of adjacent equivalent phase centers between different channels at the same time are not entirely identical. Meanwhile, different transceiver antennas also have slightly different SNRs in their measurements, which affects the quality of the 2D ISAR images.

**Figure 7:**
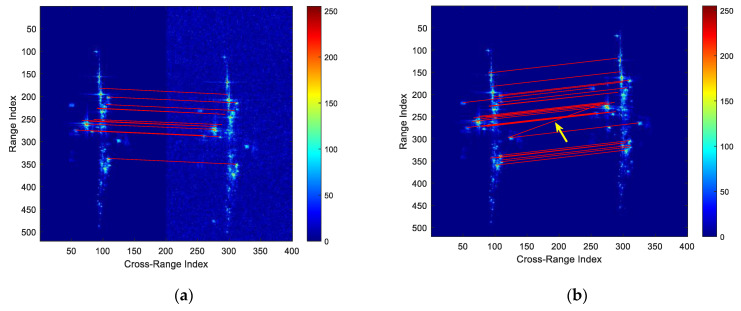
The matching results of the master image and the slave image. (**a**) The mapping relationship with a low SNR. The SNR of the slave image is 16.3 dB lower than that of the master image. (**b**) The mapping relationship with different distributions of scatters. A small number of mismatches can be seen in the matching results, as marked in the yellow arrow in (**b**).

In this paper, the channel-8 image is taken as the master image. The 16-channel 2D ISAR images are registered, and the channel-2 image and channel-9 image are taken as examples for analysis. First, the proposed method uses the SIFT algorithm for feature extraction. Figure 7 shows the matching results of the master–slave pair.

**Figure 8:**
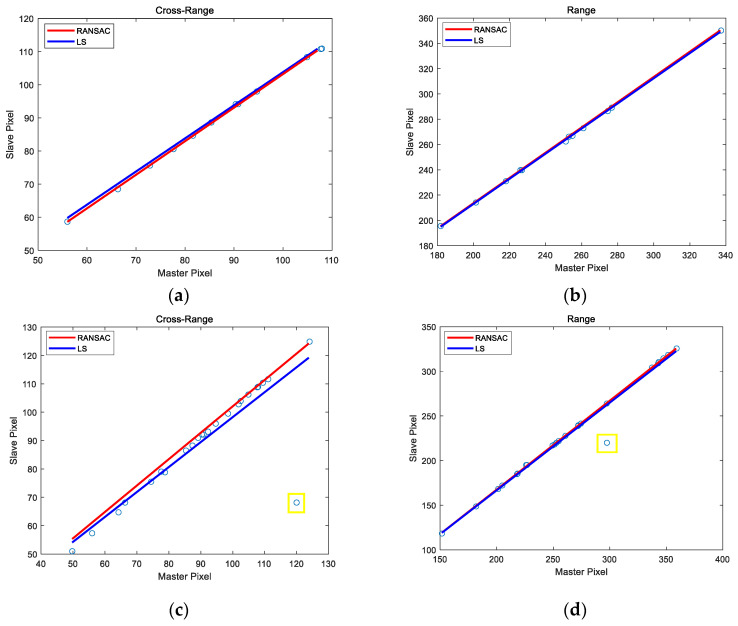
The mapping relationship of the master image and the slave image. (**a**) The mapping relationship of cross-range direction with a low-SNR image. (**b**) The mapping relationship of range direction with a low-SNR image. (**c**) The mapping relationship of cross-range direction with the distribution of scatters. (**d**) The mapping relationship of range direction with the distribution of scatters. A small amount of mismatching points is marked in the yellow box in (**c**,**d**).

After feature extraction, the RANSAC algorithm is used to reduce the influence of mismatched feature points. Compared with the least square (LS) algorithm, RANSAC obtains a robust mapping relation in building the dominant scatters model. The relative offset of coarse registration between the master image and the slave image is obtained by the dominant scatters model. Figure 8 shows the mapping relationship between the master image and the slave image.

**Figure 9:**
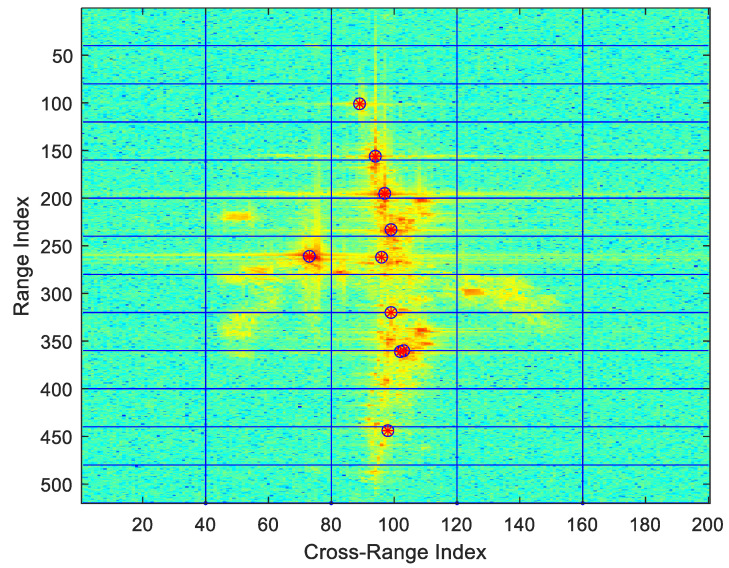
Grid backup control points of the master image.

**Figure 10:**
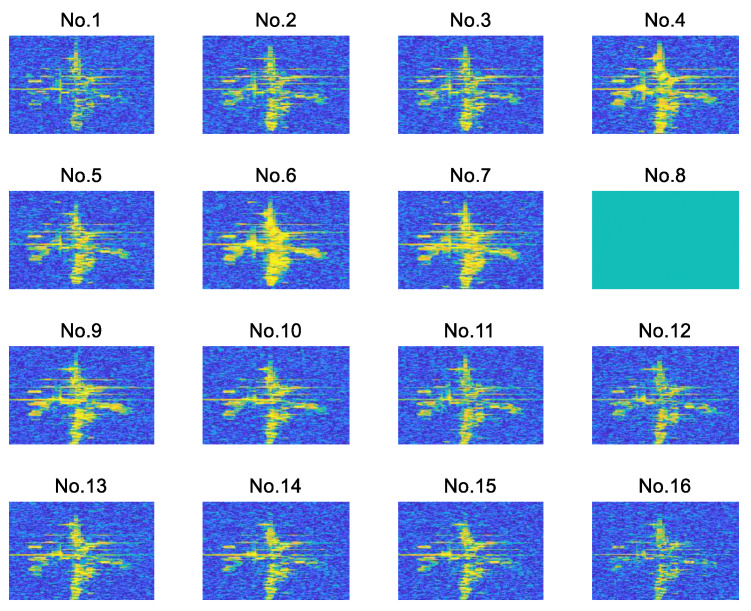
The correlation coefficient distribution of each channel.

After the master image is meshed, as shown in Figure 9 and Figure 10, dominant scatters are selected as registration control points, and the relative offset of fine registration is determined by the correlation matching method. Finally, a polynomial is used to fit the offset, and the image registration is completed by interpolating the slave image.

### 4.2. Result Analysis

The correlation coefficient of 2D ISAR images is taken as the metric to assess the image registration quality, and the registration method proposed in this paper is qualitatively analyzed. Different registration methods are used to register 16-channel 2D ISAR images. The following analysis reveals that the correlation matching method [18], max-spectrum method [19] and SAR-SIFT method [26] have poor registration performance when the SNR and distributions of scatters differ in distributed array ISAR imaging.

#### 4.2.1. Correlation Coefficients between ISAR Images

The correlation coefficient is as follows:

(17) $$\rho =\frac{E[A_{1}\times A_{2}^{*}]}{\sqrt{E\left[{\left|A_{1}\right|}^{2}\right]}\times \sqrt{E\left[{\left|A_{2}\right|}^{2}\right]}}$$

where ρ represents the correlation coefficient, $A_{1}$, $A_{2}$ are two 2D ISAR images, ${}^{*}$ denotes the complex conjugate and E[*] denotes the mathematical expectation.

Figure 10 shows the correlation coefficient distribution of each channel’s 2D ISAR image after registration with the proposed method. Remarkably, the larger the modulus, the better the registration effect between the master image and the slave image.

#### 4.2.2. Analysis and Comparison of Different Image Registration Methods

In this section, the correlation matching method, the max-spectrum method, the SAR-SIFT method and the proposed method are used to register 2D ISAR images of experimental data. The region with a correlation coefficient greater than 0.8 is regarded as the interested region for image registration, and an interval of 0.05 is used to count the number of interested region larger than the threshold. In the interested region, the larger the correlation coefficient, the better the registration quality. The ISAR image is mainly composed of a small number of scatters and a large amount of noise. In the correlation co-efficient distribution of the ISAR image, a large part of the correlation coefficient distribution is meaningless noise. Therefore, it is necessary to calculate the correlation coefficient of the region containing only scatters in the ISAR image to compare the registration effect of different methods.

**Figure 11:**
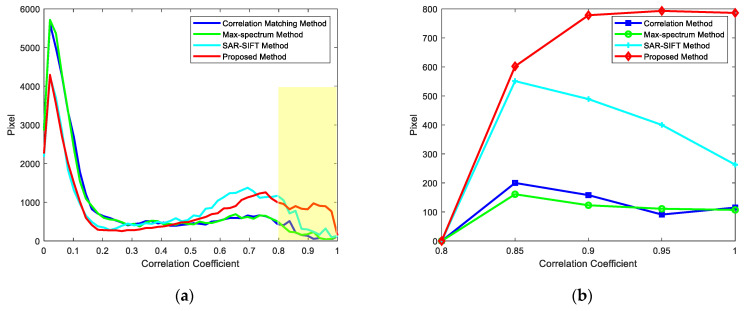
Correlation coefficients after image registration. (**a**) Correlation coefficient distribution of the channel-2 image. (**b**) Correlation coefficient distribution of the channel-2 image only containing the scatters area.

As shown in Table 3 and Figure 11, the slave image has a relatively low SNR; there is an image mismatch between the correlation matching method and the max-spectrum method, and the registration accuracy of the SAR-SIFT method is low. The number of pixels in the interested region of our method is much greater than that of the other three methods in the interested region of 0.95~1. Our proposed registration method produces more pixels with a higher correlation coefficient through the dominant scatters model and achieves better registration outcomes.

**Figure 12:**
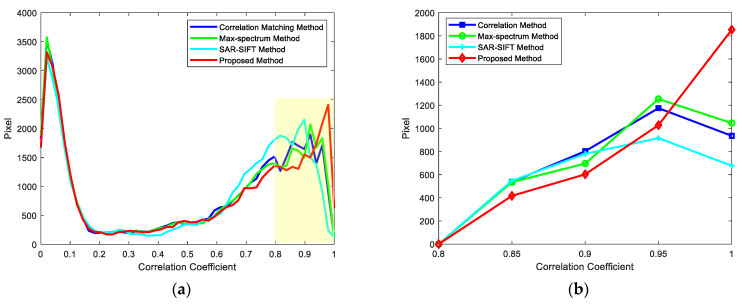
Correlation coefficients after image registration. (**a**) Correlation coefficient distribution of the channel-9 image. (**b**) Correlation coefficient distribution of the channel-9 image only containing the scatters area.

As observed in Table 4 and Figure 12, the slave image has different distributions of scatters; the peak correlation coefficient of the correlation matching method and the max-spectrum method is 0.91, and that of the SAR-SIFT method is 0.89, whereas that of the proposed method is 0.97. Furthermore, the proposed method not only has a closer to 1 peak position, but also has a great number of pixels with a higher correlation coefficient in the interested region, achieving higher registration accuracy.

It can be seen from the above analysis that the proposed registration method can achieve accurate registration for both images with a low SNR and images with different distributions of scatters. The next section will show the effect of different image registration methods on the elevation 3D reconstruction.

### 4.3. Three-Dimensional ISAR Imaging

The traditional and the proposed registration methods are used to register multiview 2D ISAR images. Based on the image registration results, the dominant scatters of the master image are selected to compensate for the amplitude and phase consistency of all 2D ISAR images.

In [15], echo signals obtained by the distributed array radar system have sparsity in the elevation dimension; they can be used for super-resolution imaging by a compressive sensing algorithm. The sparse representation of the signal $x\in R^{N}$ is as follows:

(18) $$x=\sum_{i=1}^{N}\theta_{i}\varphi_{i}=\Psi \Theta$$

where $\Psi =\left\{\varphi_{i}|i=1,2,\cdots ,N\right\}$ is the orthogonal basis matrix, $\theta_{i}=<x,\varphi_{i}>$ is the projection coefficient and $\Theta =\Psi^{T}x$ is the projection coefficient vector.

When the signal x is K−sparse in the Ψ domain, the observation matrix $A\in R^{M\times N}$ can be used to measure the sparse coefficient Θ linearly, and the observation vector $y\in R^{M}$ can be obtained as follows:

(19) y=AΘ

When the signal contains noise **e**, the observation vector becomes:

(20) y=AΘ+e

Compressed sensing is a technique for the reconstruction of sparse signals. When Θ of Equation (19) or Equation (20) is K−sparse, sparse solutions can be obtained by solving the following optimization problem:

(21) $$\underset{\Theta }{\min}{\left\|y-A\Theta \right\|}_{2}^{2}s.t.{\left\|\Theta \right\|}_{0}\leq K$$

Common reconstruction methods include the greedy tracking algorithm, the convex relaxation algorithm and the combination algorithm. The greedy tracking algorithm is widely used for its simple structure and low computation requirement. In this paper, OMP [30], one of the greedy tracking algorithms, is used to reconstruct the image sequence after amplitude and phase correction.

The OMP algorithm gradually approaches the original signal by selecting a locally optimal solution in each iteration in a greedy manner. First, the correlation principle is adopted to select the atom that best matches the iteration margin. Second, the selected atoms are Gram–Schmidt orthogonalized. Third, the signal is projected onto the space composed of these orthogonal atoms, and the component and iteration margin of the signal on the selected atoms are obtained. Finally, the residual is decomposed using the above procedure. The components and iterative residuals of the signal on the selected atom are obtained, and the residuals are decomposed using the same method. The residual is expressed as follows:

(22) $$r_{k}=y-A_{k}{\hat{\Theta }}_{k}$$

Table 5 describes the steps of the OMP algorithm.

**Figure 13:**
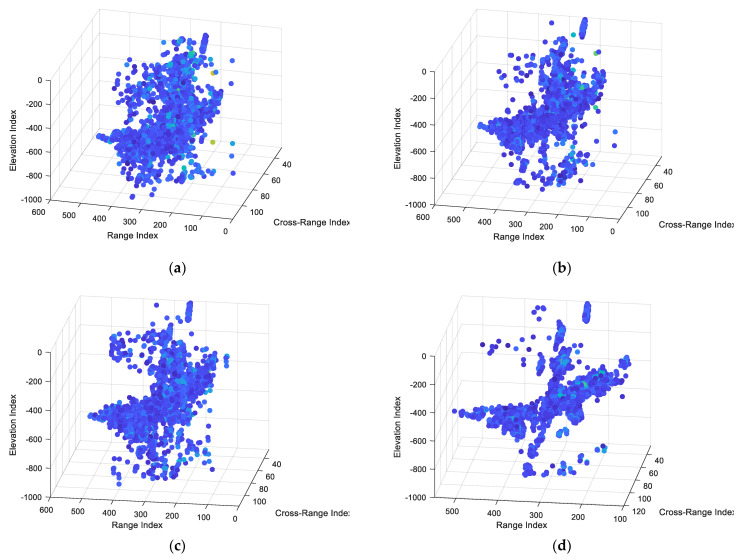
The 3D reconstruction results of 2D ISAR images with different registration methods. (**a**) Results of the correlation matching method. (**b**) Results of the max−spectrum method. (**c**) Results of the SAR−SIFT method. (**d**) Results of the proposed method.

Figure 13 shows the 3D reconstruction results, in the form of 3D point clouds, of 2D ISAR images with different registration methods using the OMP algorithm. The 3D point cloud images obtained by the proposed method contain fewer outliers and clearer features.

**Figure 14:**
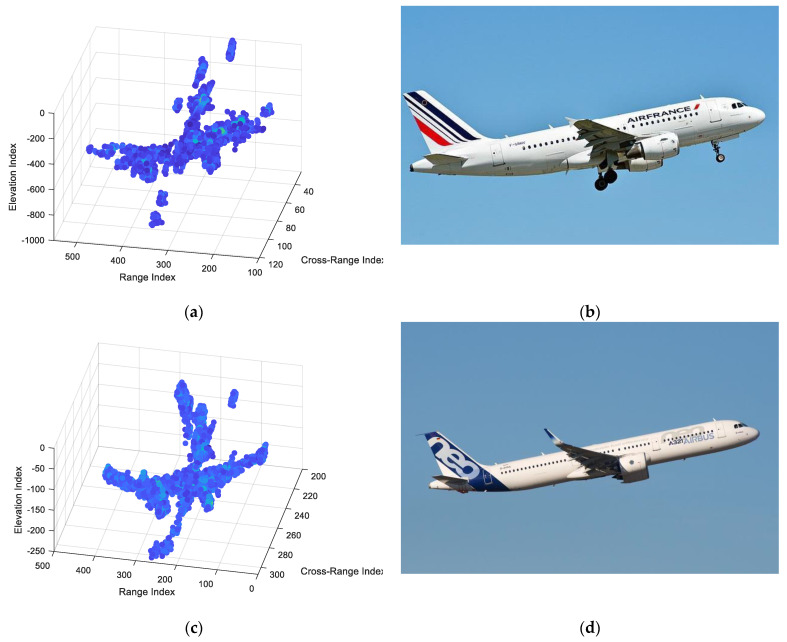
The 3D reconstruction results of the airplane with the X−band distributed array radar system. (**a**) Microwave image of Airbus A319. (**b**) Optical image of Airbus A319. (**c**) Microwave image of Airbus A321. (**d**) Optical image of Airbus A321.

Different exceptional echoes are registered with our proposed method, followed by 3D ISAR imaging. Figure 14 shows the 3D ISAR imaging filtering results of different types of the Airbus aircraft. As evident in Figure 14, the Airbus A321 has a longer fuselage than the Airbus A319, which is reflected by the different detailed features.

## 5. Conclusions

When the length of the baseline is non-negligible in the distributed array radar, the distribution of scatters is different in the 2D ISAR images. In addition, different transceiver antennas may also cause inconsistent SNRs in actual experiments. For the above reasons, the correlation matching method and the max-spectrum method have image mismatches for distributed array ISAR systems. To solve these problems, a novel image registration method is proposed that leverages feature extraction to build a dominant scatters model for coarse registration, and then uses local correlation matching for fine registration. After image registration, amplitude and phase correction is performed on the 16-channel 2D ISAR images, and the OMP algorithm is employed for super-resolution imaging in the third dimension. Compared with traditional image registration methods, the proposed method achieves better registration accuracy under the condition of a lower SNR and different distributions of scatters. The 3D reconstruction results of different aircrafts offer more detailed features, which lay the foundation for target identification. In future research, we will further explore the impact of a longer length of baseline and longer observation distance on 2D image registration and 3D reconstruction of the distributed array ISAR.

## Figures and Tables

**Table 1:** The RANSAC algorithm used to eliminate mismatching in feature extraction.

The RANSAC Algorithm Flow
1. Randomly select two points in the dataset and substitute them into the fitting equation.
2. Calculate the Euclidean distance between all matching points after and before fitting.
3. Those points with Euclidean distances less than the threshold are recorded as inliers, and the number of inliers is counted.
4. After repeating Steps 1 to 3 K times, the group with the most significant number of inliers is identified as the final fitting parameters.

**Table 2:** Configuration of the distributed array radar system.

Parameter	Symbol	Value
Carrier frequency	$f_{c}$	10 GHz
Bandwidth	$B_{\omega }$	2 GHz
Pulse repetition frequency	PRF	2.5 kHz
Reference range	$R_{\mathrm{Ref}}$	850 m
Number of APCs	Np	16
Maximum baseline	D	10.8 m

**Table 3:** The relation between pixels and correlation coefficients of the channel-2 image and the channel-8 image.

ρ	0~0.80	0.80~0.85	0.85~0.90	0.90~0.95	0.95~1
Correlation Matching Method	43619	1090	402	184	**201**
Max-Spectrum Method	43638	813	440	381	**224**
SAR-SIFT Method	40914	2362	1135	539	**546**
Proposed Method	37211	2159	2086	2273	**1767**

**Table 4:** The relation between pixels and correlation coefficients of the channel-9 image and the channel-8 image.

ρ	0~0.80	0.80~0.85	0.85~0.90	0.90~0.95	0.95~1
Correlation Matching Method	31,135	3475	4118	**4122**	2647
Max-Spectrum Method	30,907	3305	3967	**4426**	2891
SAR-SIFT Method	31,210	4524	**4700**	3860	1202
Proposed Method	29,902	3243	3409	3930	**5012**

**Table 5:** The OMP algorithm used for super-resolution imaging.

The OMP Algorithm Flow
1. Initialize $r_{0}=y$, $\Lambda_{0}=\phi$, $A_{0}=\phi$, t=1.
2. Find the index $\lambda_{t}$ with the smallest correlation coefficient: $\lambda_{t}=\arg\underset{j=1,2,\ldots ,N}{\max}\left|<r_{i-1,}a_{j}>\right|$.
3. $\Lambda_{t}=\Lambda_{t-1}\cup \left\{\lambda_{t}\right\}$, $A_{t}=A_{t-1}\cup \left\{a_{\lambda }\right\}$.
4. Find the approximate solution ${\hat{\Theta }}_{t}$ of least squares: ${\hat{\Theta }}_{t}=\arg\underset{\Theta_{t}}{\min}\left\|y-A_{t}\Theta_{t}\right\|={\left(A_{t}^{T}A_{t}\right)}^{-1}A_{t}^{T}y$.
5. Update the residual $r_{t}=y-A_{t}{\hat{\Theta }}_{t}=y-A_{t}{\left(A_{t}^{T}A_{t}\right)}^{-1}A_{t}^{T}y$.
6. t=t+1, if t≤K, return to Step 2; otherwise, stop iteration.
7. In the last iteration, ${\hat{\Theta }}_{t}$ reconstructs the non-zero term of $\Lambda_{t}$.

## Data Availability

Not applicable.
